# DermArtifactDB: a multi-label dataset for artifact annotation in public dermoscopic image repositories

**DOI:** 10.3389/fdata.2026.1866430

**Published:** 2026-10-08

**Authors:** Vanesa Gómez-Martínez, David Chushig-Muzo, Isabel Polimón-Olabarrieta, Cristina Soguero-Ruiz

**Affiliations:** 1 Department of Signal Theory and Communications, Rey Juan Carlos University, Madrid, Spain; 2 Department of Dermatology, Hospital Universitario de Móstoles, Madrid, Spain

**Keywords:** artifact detection, dermoscopic image artifacts, dermoscopy, multi-label annotation, skin lesion analysis

## 1 Introduction

Dermoscopy is a widely adopted, non-invasive imaging technique that plays a crucial role in the early detection and clinical assessment of skin lesions (Pathania et al., 2022). Its effectiveness, however, depends on the quality of the acquired images. In clinical practice, dermoscopic images often contain visual artifacts, such as hair, dark corners, gel bubbles, ruler markings, or ink markings, which are not part of the lesion itself. These artifacts can affect the visual appearance of the lesion, modifying color and texture patterns and complicating both clinical assessment and automated image analysis (Celebi et al., 2015; Barata et al., 2018; Gómez-Mart́ınez et al., 2024a,b). Consequently, artifacts are considered an important factor contributing to noise and bias in computer-aided diagnosis (CAD) systems, impacting model interpretability, robustness, and diagnostic performance (Bissoto et al., 2020; Winkler et al., 2019).

Although interest in automated dermoscopic image analysis has increased, publicly available datasets specifically focused on artifact annotation remain limited. Public datasets, including International Skin Imaging Collaboration (ISIC)-2019 (Hernández-Pérez et al., 2024; Tschandl et al., 2018), PH2 (Mendonça et al., 2013), and Derm7pt (Kawahara et al., 2018), contain images with visible acquisition artifacts, but they are primarily used to train models for skin lesion classification and do not include explicit labels for artifact presence or type. The lack of artifact annotations introduces several limitations. First, it limits the ability to study how artifacts affect model performance and interpretability. Second, it hinders the development of preprocessing methods to detect and mitigate artifacts. Third, although multiple artifacts often co-occur within the same image, existing datasets rarely capture this multi-artifact scenario, and most studies (Gómez-Mart́ınez et al., 2024a; Winkler et al., 2019, 2021; Sies et al., 2021) instead focus on individual artifacts.

To date, only a limited number of studies have addressed the annotation of dermoscopic artifacts. Bissoto et al. (2020) introduced manual artifact annotations for investigating dataset bias and spurious correlations, quantifying how artifacts act as confounding factors for lesion classification. They labeled small subsets of two dermoscopic datasets: 2,594 images from ISIC 2018 (out of more than 12,000 available images) and 872 images from the Dermoscopic Atlas (out of 1,746). While this work demonstrated the relevance of artifact-aware analysis, the annotation methodology is limited in scope, as it relies on fully manual labeling applied to small subsets of the available data. In addition, the absence of a defined and reproducible annotation procedure limits its scalability and reuse. More recently, Bissoto et al. (2022) extended this line of research by training per-artifact classifiers on previously introduced manual annotations. These classifiers were then applied to infer artifact presence in larger datasets such as ISIC-2019. Although this strategy enabled large-scale analysis, the annotations correspond to classifier output scores (probabilistic predictions) rather than verified binary ground-truth labels derived from systematic expert annotation. Moreover, these predictions are reported only for a single dataset and lack expert validation, which may lead to discrepancies with the true artifact labels, especially for difficult-to-detect artifacts.

To address these limitations, we introduce DermArtifactDB, a unified and standardized dataset that provides multi-label annotations for seven dermoscopic artifacts (hair, dark corners, gel border, gel bubbles, ruler markings, ink markings, patches). These annotations are provided for the complete image sets of three widely used public repositories (ISIC-2019, PH2, and Derm7pt), comprising 20,211 images in total (19,000 from ISIC-2019, 1,011 from Derm7pt and 200 from PH2). For each image, DermArtifactDB provides one image-level binary label per artifact category (presence/absence of each of the seven artifacts), amounting to 141,477 labels. These labels do not indicate where each artifact is located within the image, and no pixel-level segmentation masks or region annotations are provided. As the images come from these public datasets, our contribution lies not in the images themselves but in the standardized, expert-validated artifact metadata, together with the annotation pipeline and dataset-level statistics released with it. The annotations were generated using a robust data-driven pipeline that combines classical image-processing detectors, deep learning-based segmentation models, a transformer-based multi-label detector [C-Tran (Lanchantin et al., 2021)], and expert manual review with clinical oversight. This multi-phase design was developed to ensure annotation consistency and reliability across all three datasets. DermArtifactDB enriches existing dermoscopic repositories with artifact-level metadata, enabling the study of artifact distribution, diversity, and co-occurrence, and supporting the development of more transparent, interpretable, and robust CAD systems.

## 2 Materials and methods

This section describes the data sources and the multi-phase annotation framework used to construct the proposed dermoscopic artifact dataset. The methodology follows the four-phase pipeline shown in Figure 1, encompassing data collection, classical automatic artifact detection, transformer-based artifact detection, and expert manual review.

**Figure 1:**
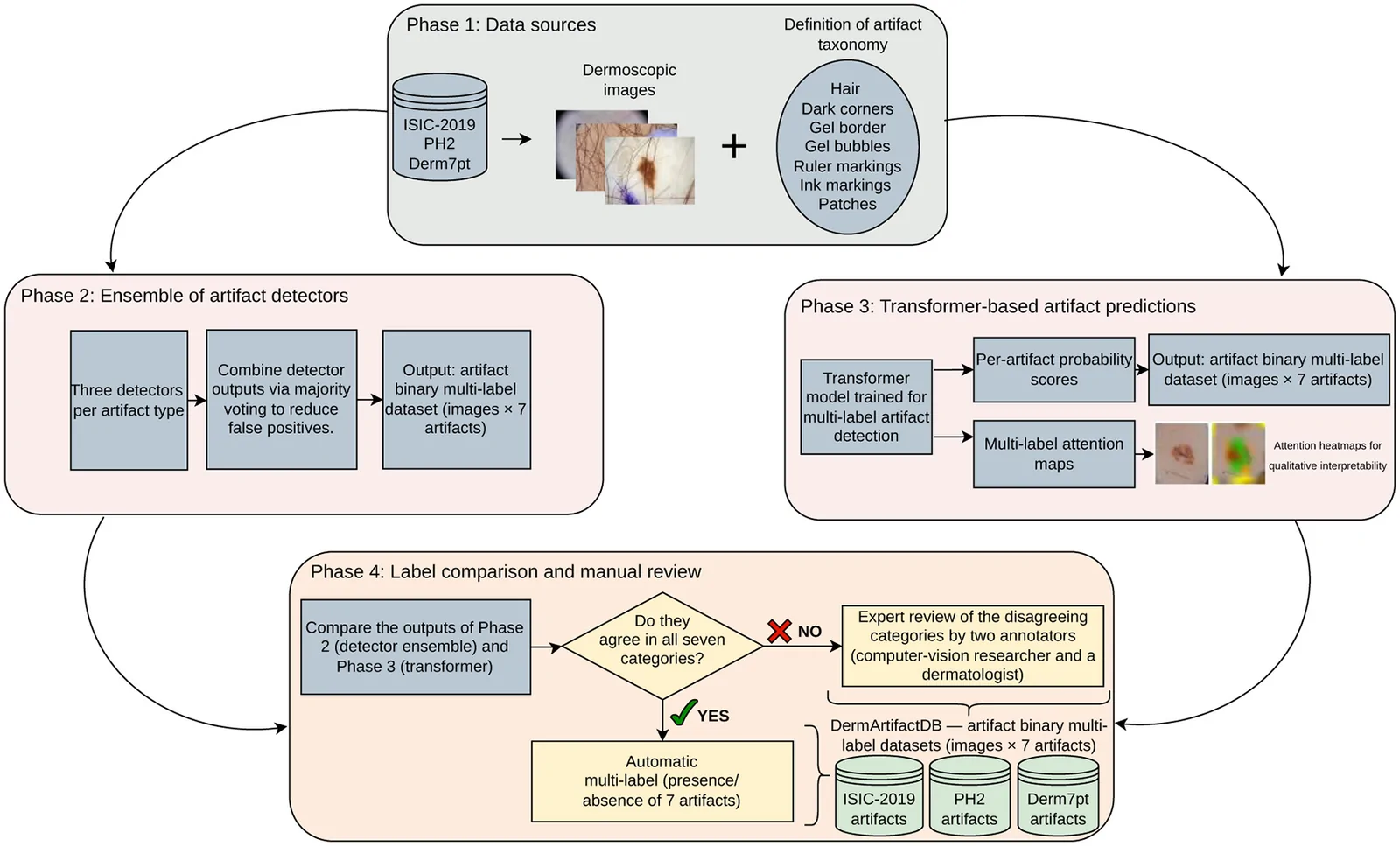
Data-driven artifact annotation pipeline for dermoscopic images. The process involves four main phases: (1) collection of public dermoscopic datasets and definition of the seven-category artifact taxonomy; (2) artifact detection using an ensemble of artifact-specific detectors; (3) transformer-based multi-label artifact detection (C-Tran); and (4) label comparison and expert manual review. The outputs of Phase 2 and Phase 3 are artifact binary multi-label datasets, with one seven-dimensional binary vector assigned to each image. The attention maps shown in Phase 3 are auxiliary visualizations used only during the expert review and are not part of the released labels. This figure is original work by the authors. The dermoscopic image thumbnails in this figure is taken from the publicly available ISIC 2019 Challenge dataset (ISIC Archive, https://challenge.isic-archive.com/data/#2019), licensed under CC BY-NC 4.0 (Tschandl et al., 2018; Hernández-Pérez et al., 2024).

The annotation pipeline relies on two independent detection stages (Phases 2 and 3 in Figure 1), followed by expert review (Phase 4 in Figure 1). Phase 2 is an ensemble of artifact-specific detectors: six of them (dark corners, gel border, gel bubbles, ruler markings, ink markings, patches) rely on classical image-processing techniques and are fully specified by the parameters reported in their respective subsections (see Sections 2.2.2–2.2.7) to ensure reproducibility. Two deep learning segmentation models are additionally used within this stage, namely an ensemble of segmentation networks for hair detection (see Section 2.2.1) and a Double U-Net that segments the skin lesion to reduce false positives in the patch detector (see Section 2.2.4). Phase 3 is an independent detector based on a C-Tran multi-label transformer (see Section 2.3) that jointly predicts the seven artifact categories. The architectures and training configurations of all learned models are detailed in full in the corresponding subsections below. The hair-segmentation code is publicly available^1^, the C-Tran detector builds on the publicly available reference implementation of Lanchantin et al. (2021)^2^, and the Double U-Net uses the implementation released by its original authors Jha et al. (2020)^3^. The classical artifact detectors are likewise publicly available^4^.

### 2.1 Data sources

The proposed artifact-annotated dataset integrates dermoscopic images from three public datasets: ISIC-2019, PH2, and Derm7pt. Table 1 summarizes the main properties of each source dataset (origin, size, acquisition conditions, original labeling and licensing).

**Table 1:** Summary of the three source datasets integrated in DermArtifactDB.

Dataset	Images	Acquisition	Original labels	License
ISIC-2019	19,000	Multiple international centers	Eight diagnostic classes; no artifact labels	CC BY-NC 4.0
PH2	200	Hospital Pedro Hispano (Portugal)	Three diagnostic classes, lesion masks; no artifact labels	Research/educational only (ADDI)
Derm7pt	1,011	Interactive Atlas of Dermoscopy	19 diagnostic classes; no artifact labels	CC BY-NC-SA 4.0

ISIC-2019 includes 19,000 dermoscopic images from international clinical institutions (with contributions from centers in Austria, Australia and Hospital Clínic de Barcelona) captured using diverse dermatoscope devices and illumination settings. Its original labels correspond to eight diagnostic categories for skin-lesion classification and contain no artifact-level information. PH2 contains 200 high-quality dermoscopic images collected under controlled acquisition conditions at the Dermatology Service of Hospital Pedro Hispanog1 (Matosinhos, Portugal), originally annotated with diagnostic labels (three categories), lesion segmentation masks, and dermoscopic criteria. Derm7pt comprises 1,011 images derived from the Interactive Atlas of Dermoscopy and annotated with d iagnostic labels (19 categories) and the seven-point checklist criteria. None of the three repositories provides labels for the presence or type of acquisition artifacts, precisely the gap addressed by DermArtifactDB.

These datasets comprise thousands of images acquired from multiple clinical centers, dermatology departments, and imaging devices, providing a heterogeneous representation of real-world dermoscopic acquisition conditions. All datasets contain fully anonymized skin lesion images, and no patient-identifiable metadata was processed. This diversity supports the development of generalized artifact-aware algorithms.

#### 2.1.1 Artifact taxonomy

Seven artifact categories were selected based on their prevalence in dermoscopic imaging and their documented impact on both clinical interpretation and CAD systems (Barata et al., 2018). Each category corresponds to a distinct visual pattern often observed in dermoscopic images:

- **Hair**: filaments that partially occlude the lesion surface and introduce texture variations (Celebi et al., 2015; Gómez-Mart́ınez et al., 2024a).
- **Dark corners**: darkened peripheral regions caused by illumination fall-off from the circular dermatoscope lens. These underexposed areas typically appear near the image edges and may partially obscure lesion boundaries (Pewton et al., 2024).
- **Gel border**: bright or translucent ring-shaped structures formed by the interaction between contact gel and the dermatoscope's illumination (Celebi et al., 2015).
- **Gel bubbles**: circular specular bubbles trapped within the gel interface, characterized by high reflectance and sharp boundaries (Celebi et al., 2015).
- **Ruler markings**: straight, high-contrast linear structures originating from measurement tools placed near the lesion (Winkler et al., 2021).
- **Ink markings**: irregular colored traces (typically blue or black) introduced during clinical examination (Winkler et al., 2019).
- **Patches**: colored adhesive markers placed on the skin near the lesion during image acquisition, varying in shape and color (Bissoto et al., 2020).

The visual descriptions above define the characteristics of each artifact category but do not, on their own, establish the criteria for considering an artifact as present. Throughout this work, an artifact category is considered present if any part of the corresponding structure is visible in the image, regardless of its size, how much of the image it occupies, or whether it overlaps the lesion. Partial visibility at the image border is sufficient, and no minimum size, length, or contrast is required. Thus, a single thin hair, a single ruler graduation, a small bubble, or a partially visible ink trace qualifies for a positive label. Table 2 summarizes, for each category, the visual definition, this inclusion criterion, and the structures most frequently confused with it. This criterion defines the released labels and was applied throughout the expert review described in Section 2.4.

**Table 2:** Annotation criteria for the seven artifact categories, including visual definition, criterion for assigning a positive label, and structures most frequently confused with each category during annotation.

Artifact	Visual definition	Inclusion criterion	Common sources of confusion
Hair	Filaments occluding the skin surface.	At least one filament visible, in whole or in part, regardless of thickness, color or length; fine vellus hair and cut fragments included.	Straight ruler edges; skin furrows and scratches; linear pigment structures within the lesion.
Dark corners	Underexposed peripheral regions caused by lens fall-off.	At least one corner or peripheral region visibly underexposed relative to the field of view, forming a continuous dark area in contact with the image border.	Heavily pigmented lesions extending to the border; dark gel borders; globally underexposed images, not labeled as dark corners.
Gel border	Bright annular meniscus of the contact gel.	A visible arc of the annular structure, even if incomplete or restricted to part of the periphery.	Dark corners, which occupy the same region with opposite polarity; large peripheral gel bubbles; specular reflection rings.
Gel bubbles	Bright circular bubbles trapped in the contact gel.	At least one clearly delimited circular or elliptical bright structure with a distinct rim; isolated small bubbles are sufficient.	Milia-like cysts and other intralesional structures appearing as small bright round bodies; diffuse glare without a rim; gel-border fragments.
Ruler markings	Straight or graduated lines from a measurement scale.	Any portion of the scale visible in the field of view, including a single graduation mark or a partial edge.	Straight dark hairs; image-border artifacts; ink lines drawn along a ruler edge.
Ink markings	Irregular blue, black or violet traces.	Any visible trace, including marks only partially visible at the image border.	Blue-white veil and blue-gray pigmentation within the lesion; dark hair; blue-tinted adhesive patches.
Patches	Colored adhesive markers on the skin.	Any portion of an adhesive marker visible, identified by a uniform saturated color and a sharp, regular boundary.	Strongly colored gel bubbles; ink markings; colored drapes or backgrounds outside the skin area.

Figure 2 shows representative examples of the seven dermoscopic artifact categories considered in this work, using images from the ISIC-2019 dataset. Each subfigure shows the typical visual appearance and variability within a given artifact class. In addition, Figure 2h highlights the co-occurrence of multiple artifacts within the same image, reflecting the multi-label nature of real-world clinical acquisitions.

**Figure 2:**
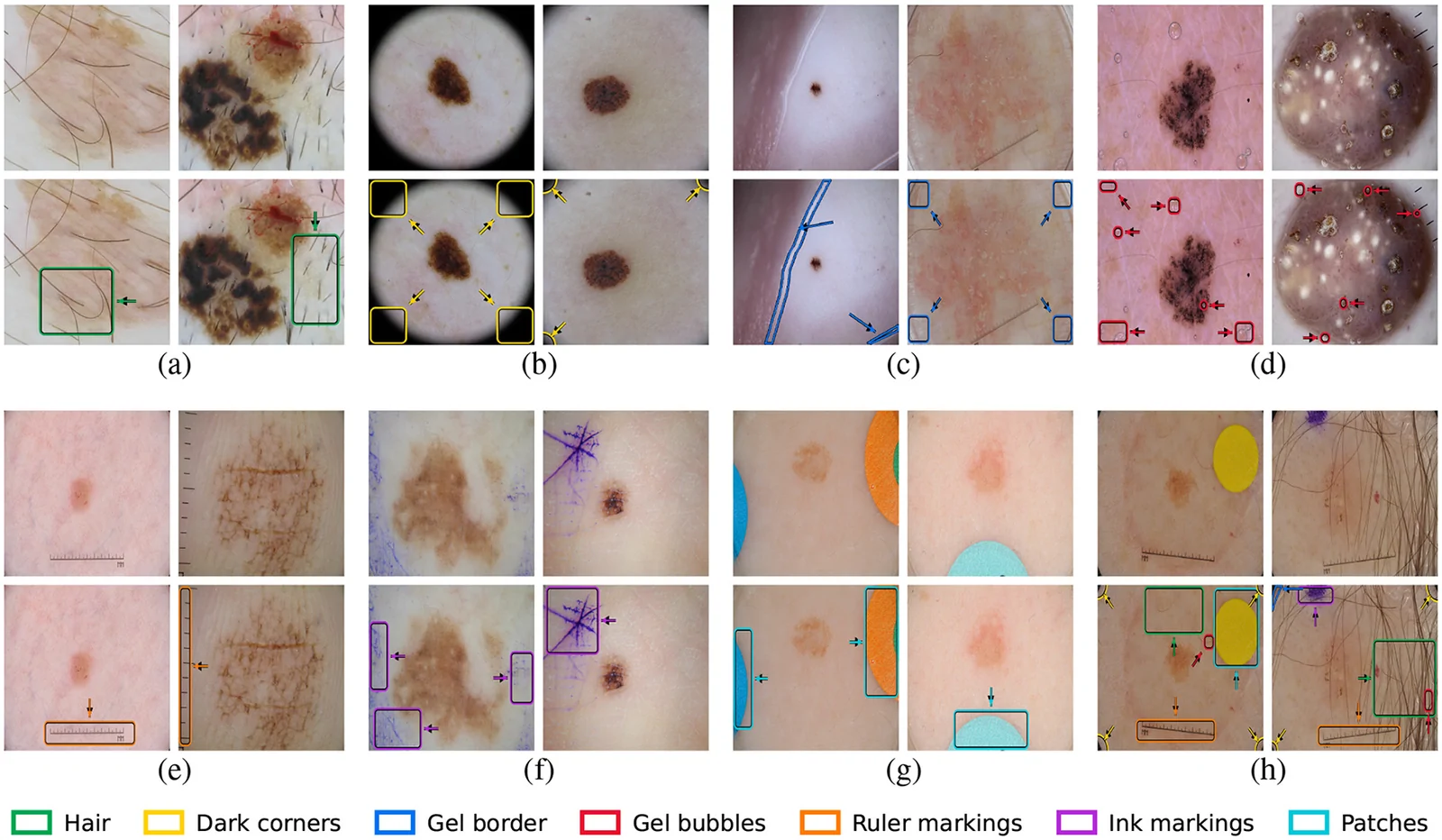
Representative examples of artifacts in dermoscopic images from ISIC-2019. Each subfigure shows two representative examples of a specific artifact type: **(a)** hair; **(b)** dark corners; **(c)** gel border; **(d)** gel bubbles; **(e)** ruler markings; **(f)** ink markings; **(g)** patches; **(h)** multiple artifacts co-occurring within a single image. Within each subfigure, the upper row shows the original images and the lower row shows the same images with the artifacts of interest indicated by colored outlines and arrows. Colors denote the artifact category, as shown in the legend, and when an artifact appears repeatedly, only representative instances are outlined. These overlays are provided for illustration only and are not part of the released annotations, which consist of image-level binary labels.

### 2.2 Ensemble of artifact detectors

This phase (Phase 2 in Figure 1) consists of artifact-specific detection modules. Six of them (dark corners, gel border, gel bubbles, ruler markings, ink markings, patches) rely on classical image-processing techniques, while hair detection employs deep learning-based segmentation models due to the morphological complexity of this artifact, as described in Section 2.2.1. This step provides an initial estimate of artifact presence in each image. For each artifact, three complementary detectors are applied in parallel, all targeting the same artifact type while capturing different aspects of its visual appearance, such as color, geometry, or intensity. This design allows the method to handle the variability in how a given artifact appears across patients, imaging devices, and acquisition conditions. For each artifact category *k*, the outputs of the three detectors $d_{1}^{\left(k\right)},d_{2}^{\left(k\right)},d_{3}^{\left(k\right)}\in \left\{0,1\right\}$ were aggregated using a majority-voting rule. An artifact was considered present when at least two of the three detectors provided a positive response; otherwise, it was considered absent. Accordingly, the output of this phase (Phase 2) is, for each image, a seven-dimensional binary vector indicating the presence or absence of each artifact category. The intermediate binary masks produced by the individual detectors are generated only as part of the detection process and are not part of the released annotations. The detectors for each artifact are defined as follows:

#### 2.2.1 Hair

Hair detection was addressed using an ensemble of three deep learning segmentation models [Attention U-Net (Oktay et al., 2018), Dense U-Net (Cai et al., 2020), and Attention Dense U-Net (Li et al., 2019)], selected to capture the high morphological variability of hair structures, following our previous hair-removal work (Gómez-Mart́ınez et al., 2024a). Each model *k*∈{1, 2, 3} produces a binary mask *M*^(*k*)^∈{0, 1}^*H*×*W*^, where *H* and *W* are the height and width of the image. Hair presence was quantified using the *hair density ratio*:


$$R_{\text{hair}}^{\left(k\right)}=\frac{1}{HW}\overset{H}{\underset{i=1}{\sum }}\overset{W}{\underset{j=1}{\sum }}M_{ij}^{\left(k\right)}$$

An image was labeled as containing hair if $R_{\text{hair}}^{\left(k\right)}>\tau_{\text{hair}}$, where τ_hair_ was empirically selected in the range [0.01, 0.05] and set to 0.03. Final predictions were obtained via majority voting across the three models.

The three networks were trained on a publicly available hair-mask dataset (500 dermoscopic images from ISIC with ground-truth hair masks), with images resized to 512 × 512, converted to the YCbCr color space and normalized to [−1, 1]. Training optimized the Dice loss using the Adam optimizer (batch size 16, up to 250 epochs with early stopping), with an 80/20 train/test split and 15% of the training data held out for validation. Full architectural and training details are provided in our previous hair-removal work (Gómez-Mart́ınez et al., 2024a).

#### 2.2.2 Dark corners

Dark corners correspond to underexposed regions located at the edges of dermoscopic images, typically caused by the dermoscopic lens geometry (Pewton et al., 2024). Detection was performed using three complementary detectors in the HSV, YCrCb, and CIELAB color spaces.

For each color space, the image was thresholded to obtain a binary mask highlighting low-luminance pixels near the image borders. The image was then divided into four quadrants: top-left, top-right, bottom-left, and bottom-right. For each quadrant, rows and columns along the image borders were analyzed. For every border row or column, we computed the proportion of pixels classified as dark:


$$f_{\text{border}}=\frac{\text{number of dark pixels in row/column}}{\text{number of pixels in row/column}}$$

A border region was considered dark if *f*_border_ ≥ τ_border_. The threshold τ_border_ was empirically selected by evaluating values in the range [0.005, 0.05], with the final value set to 0.01. This choice allowed detection of subtle vignetting effects while remaining robust to noise. The final label was obtained by majority voting across the three color-space detectors.

#### 2.2.3 Ruler markings

Ruler markings were detected by identifying straight line segments using the probabilistic Hough transform (Duda and Hart, 1972). To account for variability in ruler appearance and image resolution, three complementary detectors were used with different parameter configurations, including accumulator thresholds in the range [50, 150] and minimum line lengths in [10, 25] pixels. The final configurations were set to accumulator thresholds of 50, 100, and 150, and minimum line lengths of 10, 15, and 25 pixels for the three detectors, respectively. The probabilistic Hough transform represents each detected line as a finite segment defined by two endpoints, (*x*_1_, *y*_1_) and (*x*_2_, *y*_2_). This formulation is appropriate for dermoscopic images, where the straight edge of a ruler typically appears as multiple fragmented segments rather than as a single continuous line. Due to the rigid linear structure of a ruler, all fragments belonging to the same ruler are expected to share a common orientation. The orientation of each segment was therefore computed as follows:


$$\theta =\arctan\left(\frac{y_{2}-y_{1}}{x_{2}-x_{1}}\right)$$

The dominant direction θ^*^ was estimated as the most common orientation among the detected segments, and only segments satisfying $|\theta -\theta^{*}|<\tau_{\text{tol}}$ were retained. The tolerance parameter was selected in the range [5°, 15°], with $\tau_{\text{tol}}=10^{•}$. A positive detection was assigned if at least one set of aligned segments was identified, and final labels were obtained via majority voting.

#### 2.2.4 Patches

Patches were detected using three color spaces (HSV, YCrCb, and CIELAB). These detectors were designed to capture the warm (yellow, orange, red) and cool (green, blue) chromatic tones that are characteristic of adhesive markers. To reduce false positives, lesion regions were first segmented using Double U-Net (Jha et al., 2020) and excluded from the analysis. For lesion segmentation, we used the Double U-Net model provided by its original authors (Jha et al., 2020), pre-trained for skin-lesion segmentation. It is applied only to exclude lesion pixels from the patch analysis and was not retrained in this work.

Adhesive markers often exhibit heterogeneous chromatic appearance, combining highly saturated regions with less saturated areas due to illumination variability. To account for this, each detector generated two chromatic masks: a *low-threshold mask* capturing pixels within a broad color range (including weakly saturated regions), and a *high-threshold mask* retaining only strongly saturated pixels. Let *N*_low_ and *N*_high_ denote the number of non-zero pixels in the low- and high-threshold masks, respectively. We defined the following *color consistency ratio*:


$$r_{patches}=\frac{N_{\text{high}}}{N_{\text{low}}}\times 100$$

A patch was considered present when both color consistency and spatial extent criteria were satisfied:


$$r_{patches}>\tau_{\text{solidity}}\;\text{and }N_{\text{high}}>\tau_{\text{size}}$$

The thresholds were selected empirically (τ_solidity_∈[30, 70], τ_size_∈[500, 2000]), with final values τ_solidity_ = 50 and τ_size_ = 1250, which were consistently applied across all three color-space detectors. Final labels were obtained via majority voting across the three detectors.

#### 2.2.5 Gel border

The gel border appears as a bright, ring-shaped structure near the edges of dermoscopic images. Detection was performed using three color spaces (HSV, YCrCb, and CIELAB), focusing on peripheral regions by masking the image center to avoid confusion with circular skin lesions. Candidate circular structures were identified using the Hough transform, retaining only those whose centers lay within ±10% of the image midpoint, reflecting the typically centered position of the gel border.

For each detected circle with radius *r*_*c*_, the gel border was modeled as an annular region of width τ_width_. Two binary masks were defined: an inner mask *M*_inner_, corresponding to a circle of radius *r*_*c*_, and an outer mask *M*_outer_, corresponding to a circle of radius *r*_*c*_+τ_width_. The gel border mask was then defined as:


$$M_{\text{gel}}=M_{\text{outer}}-M_{\text{inner}}$$

To distinguish the gel border from dark corners, detections with low luminance inside the circle were discarded. A positive detection was assigned if the annular region exceeded a minimum area threshold τ_area_. Parameters were selected empirically (τ_width_∈[15, 50], τ_area_∈[1000, 5000]), with final values τ_width_ = 30 and τ_area_ = 3000. Final labels were obtained via majority voting across the three detectors.

#### 2.2.6 Gel bubbles

Gel bubbles were detected as small, approximately circular bright structures within the contact gel. Preprocessing steps [grayscale conversion, Canny edge detection (Canny, 2009), and morphological filtering] were applied to obtain clean contours, while suppressing hair regions using the hair mask (obtained as described in Section 2.2.1). Extracted contours were characterized by their area *A* and perimeter *P*, and a circularity index was computed as:


$$C=\frac{4\pi A}{P^{2}}$$

Contours with area *A* in the range [50, 1000] pixels and sufficiently high circularity *C* were retained. Three detectors with increasing circularity thresholds (*C* > 0.3, *C* > 0.4, and *C* > 0.5) were used to account for variability in bubble shape. A positive detection was assigned when at least one contour satisfied both the area and circularity constraints. Final labels were obtained via majority voting across the three detectors.

#### 2.2.7 Ink markings

Ink markings, typically characterized by blue or bluish-black tones, were detected across three color spaces (HSV, YCrCb, CIELAB). They often exhibit variability in color intensity due to illumination effects. To account for this, each detector generated two complementary masks: a *low-threshold mask* capturing a broad range of blue-like tones and a *high-threshold mask* retaining only strongly saturated or high-intensity blue pixels. Let $N_{\text{low}}^{\text{ink}}$ and $N_{\text{high}}^{\text{ink}}$ denote the number of non-zero pixels in these masks. A chromatic consistency ratio was defined as:


$$r_{\text{ink}}=\frac{N_{\text{high}}^{\text{ink}}}{N_{\text{low}}^{\text{ink}}}\times 100$$

A positive detection was assigned when both chromatic consistency and spatial extent constraints were satisfied:


$$r_{\text{ink}}>\tau_{\text{homogeneity}}\;\text{and }N_{\text{high}}^{\text{ink}}>\tau_{\text{size}}$$

Here, τ_homogeneity_ controls the required proportion of strongly saturated pixels, while τ_size_ enforces a minimum number of detected pixels. Parameters were selected empirically (τ_homogeneity_∈[30, 60], τ_size_∈[100, 500]), with final values τ_homogeneity_ = 40 and τ_size_ = 300. The same threshold values were used for the HSV, YCrCb, and CIELAB detectors. Final labels were obtained via majority voting across the three detectors.

### 2.3 Transformer-based artifact detection

We complement classical image-processing detectors with a C-Tran-based multi-label Transformer (Lanchantin et al., 2021). Building on our previous work (Gómez-Martínez et al., 2025), we adapt this model for dermoscopic artifact detection using masked label training and an imbalance-aware, Class-Adaptive Focal with Confidence (CAFC) penalty loss to capture inter-artifact dependencies and improve sensitivity to rare artifacts. This loss has been shown to outperform standard binary cross-entropy and focal loss formulations, particularly for visually subtle and infrequent artifacts.

The network jointly predicts the presence of seven artifact categories within a multi-label classification framework. C-Tran integrates a convolutional visual backbone with a Transformer encoder that models inter-label dependencies. The backbone produces a spatial feature map that is combined with a set of learnable label embeddings. During training, a masked label strategy is used, where a random subset of labels is hidden to encourage the model to infer missing annotations from contextual relationships (e.g., co-occurrence of gel border and gel bubbles).

Following our previous work (Gómez-Martínez et al., 2025), the C-Tran backbone is a ResNet-101 network initialized with ImageNet-pretrained weights and fine-tuned jointly with the Transformer encoder. The model is configured with an input resolution of 500 × 500, three Transformer layers, four attention heads, and a batch size of 64. The model was trained on the ISIC-2019 dataset (80% training / 20% test, with 15% of the training data used for validation, repeated over five random seeds) using the CAFC loss of (Gómez-Martínez et al., 2025) (γ = 1.0, α = 0.5, λ = 0.05). We used the Adam optimizer with a learning rate of 10^−4^, a ReduceLROnPlateau scheduler (factor 0.1, patience 3), and early stopping (patience 15 epochs) based on the validation loss. Our implementation builds on the publicly available reference code of C-Tran (Lanchantin et al., 2021).

Although transformers typically require large training sets, C-Tran mitigates this through the ImageNet-pretrained backbone and transfer learning described above, together with the masked-label strategy and the CAFC loss. This allows it to be trained effectively on the available data despite the comparatively low inductive bias of transformers. Furthermore, C-Tran was compared against CNN baseline models (ResNet-101, DenseNet-121 and EfficientNet-B0) under the same five random train/test splits and evaluation protocol on ISIC-2019. Performance was assessed with four standard multi-label metrics: the mean Average Precision (mAP), the micro-averaged overall F1-score (OF1) and overall recall (OR), and the macro-averaged class-wise F1-score (CF1). C-Tran achieved the best result on all of them (see in Supplementary Table S1).

The model outputs a vector of seven independent per-class probabilities:


$$\text{p}=\left(p_{1},p_{2},\ldots ,p_{7}\right),\;p_{k}\in \left[0,1\right],$$

where each *p*_*k*_ is obtained through a class-wise sigmoid activation and denotes the probability that artifact *k* is present, independently of the other classes. These values do not form a probability distribution and are not constrained to sum to one (in contrast to the softmax output of a single-label classifier). Binary predictions are obtained by thresholding each probability at 0.5:


$${ŷ}^{\left(k\right)}=\{\begin{array}{cc} 1, & p_{k}\geq 0.5\\ 0, & p_{k}<0.5 \end{array}$$

The output of this phase (Phase 3) is also a seven-dimensional binary vector, obtained by thresholding the per-class probabilities at 0.5. Thus, both phases produce the same type of output (image-level binary labels), and neither generates artifact region masks. The multi-label attention maps generated by C-Tran are not used as annotations; they serve only as a visual aid for the expert reviewers during Phase 4 (see Section 2.4) and are additionally released for qualitative interpretability analysis (see Section 3).

### 2.4 Label comparison, expert manual review, and dataset consolidation

Artifact predictions were generated independently using the two approaches described in the previous sections: the classical ensemble detector and the C-Tran model. Predictions were compared at the image level and across individual artifact categories to identify discrepancies. Labels were accepted for each artifact category on which the two approaches produced identical predictions. Categories with discrepant predictions were flagged for expert review.

We first note that the released annotations are image-level binary labels; DermArtifactDB does not provide region masks or bounding boxes. Consequently, the expert review determined, for each image and each artifact category, a single binary decision (presence/absence) rather than a spatial region, and the reference labels correspond to these consolidated binary decisions.

Images with disagreement in at least one artifact category were flagged for further review (the discrepancy criterion) and subsequently examined by two independent annotators: a computer vision researcher and a board-certified dermatologist. The computer-vision researcher assessed the technical and visual evidence for each artifact—whether a detected structure is consistent with the visual definition of the artifact—using the original image together with the C-Tran attention maps as a spatial guide. The dermatologist, in turn, provided clinical validation, judging artifact presence in the context of real dermoscopic acquisition. For each case, the experts received: (i) the original dermoscopic image; (ii) the predictions from the ensemble approach; (iii) the predictions generated by C-Tran; and (iv) the multi-label attention maps produced by C-Tran, which highlight the image regions contributing to each prediction. Based on this information, both annotators independently assigned binary labels for each of the seven artifact categories.

The review of each flagged image followed an explicit protocol. Using the visual definitions established in the artifact taxonomy (see Section 2.1.1) as the decision criterion, the annotators proceeded as follows: (1) the original dermoscopic image was inspected at full resolution; (2) the two automated predictions (classical ensemble and C-Tran) were compared to identify the artifact categories in disagreement; (3) for each of these categories, the corresponding C-Tran attention map was used to locate the image evidence supporting the prediction; and (4) a binary presence/absence label was assigned to that category against its taxonomy definition. The categories on which both approaches already agreed retained the agreed label and were not re-annotated.

Discrepancies were resolved as follows. When the two automated approaches disagreed, but the two annotators agreed with each other, the annotators' consensus was adopted. When the two annotators disagreed with each other (including cases in which both differed from the automated outputs), the case was discussed jointly until consensus was reached. In the rare event that consensus was not reached, the dermatologist's assessment was adopted as the reference standard. For the PH2 dataset, all images were reviewed, and every discrepancy was resolved through joint discussion.

Before any joint discussion, the two annotators assigned labels independently to all 10,711 artifact-level decisions submitted for review. Overall percentage agreement was 94.3%, and Cohen's kappa (κ) (Cohen, 1960) was 0.88. Agreement was highest for patches (κ = 1.00), ruler markings (κ = 0.98), and dark corners (κ = 0.96), and lowest for gel border (κ = 0.69) and gel bubbles (κ = 0.76), the categories most readily confused with each other and, in the case of gel bubbles, with intralesional structures of similar appearance. A total of 609 decisions (5.7%) required joint discussion before consensus was reached, of which 76% corresponded to those two categories. Per-category results are reported in Supplementary Table S2.

The annotators had access to the automated predictions and to the attention maps throughout the review, which may influence their decisions toward confirming those predictions. This risk was reduced by requiring every decision to be based on the original image at full resolution, and by the fact that, in most reviewed cases, the two automated approaches had produced conflicting predictions. For PH2, the artifact labels were produced by expert manual annotation carried out independently of this pipeline and therefore provide a reference that is not affected by this limitation. For the remaining datasets, this procedure cannot fully exclude confirmation bias, which remains a limitation of the annotation process.

Therefore, the final dataset consists of consolidated labels obtained through the pipeline described above, comprising the ensemble of classical detectors (Phase 2), transformer-based detection (Phase 3), and independent expert review (Phase 4).

## 3 Artifact-annotated dataset description

The released dataset provides standardized multi-label annotations of dermoscopic artifacts for images from three publicly available repositories: ISIC-2019, PH2, and Derm7pt. Due to licensing constraints, the dermoscopic images are not redistributed. Instead, interested readers can obtain the original images directly from the corresponding source repositories ^5^ and match them with the corresponding artifact annotations using their respective unique image identifiers (e.g., filenames or ISIC identifiers).

In the repository structure of the artifact annotation dataset (DermArtifactDB) ^6^, a directory named annotations/ contains one CSV file per dataset (ISIC-2019, PH2, and Derm7pt), each providing binary artifact labels for the corresponding images. An optional directory named heatmaps/ is also provided, organized into dataset-specific subdirectories containing attention-based visualization images generated by C-Tran. These heatmap visualizations highlight image regions that contributed most strongly to each artifact prediction and are provided for qualitative inspection and interpretability analysis. They are not used as annotations and are independent of the released binary artifact labels. A directory named metadata/ includes the summary_statistics.csv file, which reports dataset-level statistics, including: (i) artifact prevalence, (ii) the distribution of the number of artifacts per image, and (iii) representative pairwise artifact co-occurrence rates. A README.md file provides an overview of the annotation pipeline, the dataset structure, and instructions for linking the annotation files to the original images. In addition, to follow community standards and facilitate reuse, DermArtifactDB is documented following the *Datasheets for Datasets* framework (Gebru et al., 2021) and adheres to the FAIR principles for scientific data management (Wilkinson et al., 2016). The complete datasheet is provided as Supplementary material.

To characterize the structure and composition of the annotated datasets, Figure 3 summarizes artifact prevalence and co-occurrence patterns across ISIC-2019, PH2, and Derm7pt. Figure 3a reports the prevalence of each artifact category in the three datasets, defined as the proportion of images in which the category is present. Figures 3b–d show the distribution of the number of artifacts per image in ISIC-2019, PH2, and Derm7pt, respectively, indicating how many images contain zero, one, two, or multiple artifact types. These histograms provide an overview of overall artifact burden and highlight the prevalence of images containing multiple artifact types within each dataset. Finally, Figures 3e–g display pairwise co-occurrence matrices computed at the dataset level. Consider a dataset of *N* images and seven artifact categories, indexed by *a* and *b*, and let *y*_*a, i*_, *y*_*b, i*_∈{0, 1} take the value 1 when the corresponding artifact is present in image *i* and 0 otherwise. The co-occurrence matrix is the 7 × 7 matrix whose entry $C_{a,b}=\left(1/N\right)\underset{i}{\sum }y_{a,i}y_{b,i}$ is the number of images in which artifacts *a* and *b* are simultaneously present, divided by the total number of images in the dataset. Every entry, on and off the diagonal, therefore has the same denominator, and the matrix is symmetric, since *C*_*a, b*_ = *C*_*b, a*_. Off-diagonal entries give the proportion of images containing both artifacts, and the diagonal entry reduces to the proportion of images containing a given artifact, that is, its prevalence, which coincides with the value shown in Figure 3a. This normalization differs from one over artifact instances, in which each artifact is expressed as a share of the total number of annotated instances rather than of the number of images; the two coincide only when every image contains exactly one artifact.

**Figure 3:**
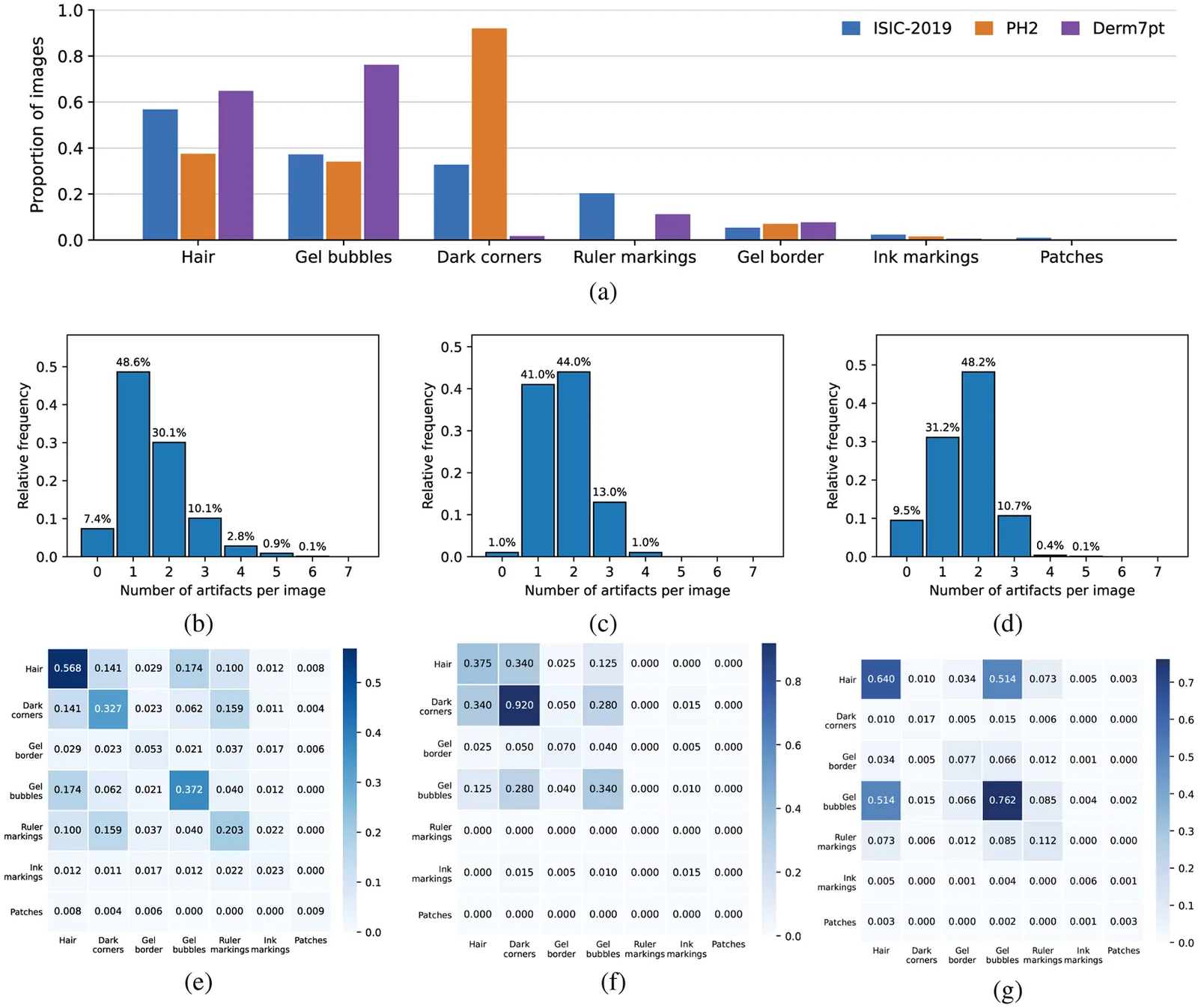
Summary visualizations of artifact distributions in the ISIC-2019, PH2, and Derm7pt artifact datasets. **(a)** Prevalence of each artifact category in the three datasets, corresponding to the diagonal entries of the co-occurrence matrices. **(b–d)** Relative frequency distribution of the number of artifacts per image in ISIC-2019, PH2, and Derm7pt, respectively; **(e–g)** artifact co-occurrence matrices for the same datasets, each entry normalized by the number of images in the dataset.

Clear differences are observed across datasets in both the number and type of artifacts. ISIC-2019 exhibits the highest diversity of artifact types and frequent multi-artifact configurations. Hair is present in 56.8% of its images, gel bubbles in 37.2% and dark corners in 32.7%, and it is the only dataset in which ruler markings are frequent (20.3%), reflecting its heterogeneous and large-scale acquisition setting. In contrast, PH2 shows a more constrained artifact distribution, dominated by dark corners, which appear in 92.0% of its images, followed by hair (37.5%) and gel bubbles (34.0%); ruler markings and patches are absent. Derm7pt presents a distinct pattern characterized by a high prevalence of gel-related artifacts, particularly gel bubbles (76.2%), together with a substantial presence of hair (64.8%) and an almost complete absence of dark corners (1.7%), highlighting the influence of dataset-specific acquisition conditions. Despite these differences, all three datasets exhibit artifact co-occurrence, indicating that certain artifacts tend to co-occur more frequently than others.

Table 3 summarizes the frequency of artifact-level annotation disagreements across datasets, where a disagreement is defined as a mismatch between the outputs of the classical detectors and the transformer-based model for a given artifact. The proposed hybrid annotation framework required dataset-dependent levels of manual review. In ISIC-2019, 8,925 artifact-level decisions (out of 133,000 total, corresponding to 6.71%) required manual review due to disagreement between the classical detectors and the transformer model. In Derm7pt, 386 artifact-level decisions (out of 7,077 total, 5.45%) required review. All discrepant cases were independently examined by two annotators—a computer-vision researcher and a dermatologist—and consensus was reached through discussion in all instances.

**Table 3:** Summary of artifact-level disagreement statistics across the ISIC-2019, Derm7pt, and PH2 datasets.

Artifact	ISIC-2019 (n)	ISIC-2019 (%)	Derm7pt (n)	Derm7pt (%)	PH2 (n)	PH2 (%)
Total artifact-level decisions	133,000	100%	7,077	100%	1,400	100%
Total discrepancies	8,925	6.71%	386	5.45%	57	4.07%
Discrepancies by artifact type						
Hair	1,928	1.45%	160	2.26%	22	1.57%
Dark corners	2,438	1.83%	12	0.17%	8	0.57%
Gel border	434	0.33%	0	0%	5	0.36%
Gel bubbles	2,705	2.03%	147	2.08%	20	1.43%
Ruler markings	1,268	0.95%	63	0.89%	—	—
Ink markings	148	0.11%	3	0.04%	2	0.14%
Patches	4	0.003%	1	0.01%	—	—

The table reports the total number of decisions, the total discrepancies, and their distribution by artifact type (hair, dark corners, gel border, gel bubbles, ruler markings, ink markings, and patches), expressed in both absolute counts (n) and percentages (%).

To further assess the robustness of the proposed methodology, the PH2 dataset (200 images, 1,400 artifact-level decisions) underwent a complete manual review. All artifact labels, including both agreement and disagreement cases, were examined by the same annotators, and any discrepancies were resolved through discussion. This exhaustive evaluation provides a fully expert-reviewed reference for that dataset, against which the automated components of the pipeline can be evaluated directly. Additionally, of the labels on which the two automated approaches agreed, a random subset corresponding to approximately 3% of the images was inspected manually (570 images in ISIC-2019 and 30 in Derm7pt, i.e., 3,990 and 210 artifact-level decisions, respectively). Each of these decisions was compared with the judgment of the two annotators on the image itself. A decision was recorded as a false positive when the label indicated the artifact as present, and the annotators judged it absent, and as a false negative when the label indicated it as absent, and the annotators judged it present. In ISIC-2019, 110 artifact-level decisions were corrected (2.76%), comprising 71 false positives and 39 false negatives. In Derm7pt, seven decisions were corrected (3.33%), comprising five false positives and two false negatives. Corrections were mainly associated with gel bubbles, dark corners, and hair, whereas patches and ink markings required very few. Per-category results are reported in Supplementary Table S3.

Because the PH2 artifact labels were produced independently of the annotation pipeline, they allow the two automated approaches to be evaluated directly against an external reference. Sensitivity, specificity, precision, F1-score, and Cohen's kappa for the classical detector ensemble and for C-Tran, computed separately for each artifact category over the 1,400 artifact-level decisions in PH2, are reported in Supplementary Table S4. Both approaches perform well on the more frequent, high-contrast categories and less well on the less frequent ones. These results should be interpreted in the context of the class distribution in PH2, which is markedly imbalanced for several categories: dark corners are present in 184 of the 200 images, leaving only 16 negative instances, whereas ink markings occur in only 3. For such categories, a small number of individual errors can substantially affect the corresponding metrics, and specificity and precision should therefore be interpreted alongside the absolute instance counts rather than in isolation.

Although this approach substantially reduces labeling errors, some subtle artifacts—such as very small or partially visible structures—may still be difficult to detect. Moreover, agreement between the two automated approaches does not guarantee correctness, since both may fail in the same way on visually subtle artifacts or on recurrent confounders, and such cases are by construction invisible to the disagreement statistic. The disagreement rate reported in Table 3 therefore does not provide a direct estimate of the annotation error rate, as it cannot detect the cases in which both methods make the same error. On PH2, 74 of the 1,343 artifact-level decisions on which the two approaches agreed (5.51%) were inconsistent with the expert reference (Supplementary Table S3), a rate of the same order of magnitude as the 4.07% disagreement observed on that dataset. Undetected annotation errors may therefore remain mainly in ISIC-2019 and Derm7pt, where the labels agreed upon by both approaches were not individually reviewed beyond the random subset described above. Consequently, the resulting annotations should not be interpreted as absolute ground truth, but rather as a high-quality reference dataset suitable for systematic analysis and benchmarking.

A final consideration concerns the granularity of the annotations. DermArtifactDB provides image-level binary labels indicating, for each image, whether each of the seven artifact categories is present, and does not include bounding boxes, segmentation masks, instance counts, or measures of artifact area or intensity. This design supports the analyses reported here, the training and evaluation of image-level multi-label classifiers, and the selection or stratification of subsets according to artifact composition, and it makes expert-supervised annotation feasible at the scale of the three repositories. However, it does not support tasks that require artifacts to be localized within the image, such as artifact segmentation, detection, inpainting-based removal, or the quantification of the affected image area. It also means that the labels do not distinguish images in which an artifact is dominant from those in which it is barely visible, since both receive the same label. The prevalence and co-occurrence statistics reported above should therefore be interpreted as the proportion of images in which each artifact is present, rather than as a measure of its extent. Region-level annotation of a representative subset of the images, providing masks or bounding boxes for the seven categories, constitutes the natural extension of this resource and is left as future work.

## Data Availability

The datasets presented in this study can be found in online repositories. The names of the repository/repositories and accession number(s) can be found below: https://doi.org/10.5281/zenodo.18324962.

## Footnotes

1 https://github.com/ai4healthurjc/hair-removal-aotgan-skin-lesions.

2 https://github.com/QData/C-Tran.

3 https://github.com/DebeshJha/Doubleunet_pytorch.

4 https://github.com/ai4healthurjc/dermartifactdb-classical-detectors.

5 The original datasets are available at: ISIC-2019 (https://challenge.isic-archive.com/data/#2019), PH2 (https://www.fc.up.pt/addi/ph2%20database.html), and Derm7pt (https://derm.cs.sfu.ca/Welcome.html).
