# Supplementary material for: DermArtifactDB: a multi-label dataset for artifact annotation in public dermoscopic image repositories
Source: Front Big Data. 2026 Oct 8;9:1866430. doi: 10.3389/fdata.2026.1866430 (PMC13686860; doi:10.3389/fdata.2026.1866430)
Supplement: Supplementary file 1 [file Data_Sheet_1.pdf]

Supplementary Material for the paper: *DermArtifactDB: A multi-label dataset for artifact annotation in public dermoscopic image repositories*

Vanesa Gómez-Martínez [1,*], **David Chushig-Muzo** [1], **Isabel Polimón-Olabarrieta** [2] **and Cristina Soguero-Ruiz** [1,*]

[1] *Department of Signal Theory and Communications, Rey Juan Carlos University, Fuenlabrada, Madrid, Spain*
[2] *Department of Dermatology, Hospital Universitario de Móstoles, Móstoles, Madrid, Spain*

Correspondence*:
Corresponding Author
vanesa.gomez@urjc.es; cristina.soguero@urjc.es

This document provides the Supplementary Material for the above paper. It contains: *(i)* a datasheet documenting the dataset (Section 1); *(ii)* a comparison between C-Tran and CNN baselines (Section 2); *(iii)* the inter-annotator agreement statistics (Section 3); *(iv)* the results of the manual validation of the automatically accepted labels (Section 4); and *(v)* the evaluation of the two automated detection approaches on PH2 (Section 5).

1 DATASHEET

The dataset is documented following the *Datasheets for Datasets* framework (1). It also adheres to the FAIR principles for scientific data management (2).

1.1 Motivation

For what purpose was the dataset created?
DermArtifactDB was created to provide standardized, expert-validated, image-level annotations of acquisition artifacts (seven categories) for three widely used public dermoscopic repositories (ISIC-2019, PH2 and Derm7pt) that lack such labels. It enables the study of artifact prevalence, diversity and co-occurrence, the development of artifact detection and mitigation (preprocessing) methods, and the investigation of how artifacts affect the robustness, interpretability and fairness of computer-aided diagnosis (CAD) systems.

Who created the dataset and on behalf of which entity?
The dataset was created by V. Gómez-Martínez, D. Chushig-Muzo and C. Soguero-Ruiz (Department of Signal Theory and Communications, Rey Juan Carlos University, Madrid, Spain). V. Gómez-Martínez, as the computer-vision researcher, carried out the technical artifact annotation, and I. Polimón-Olabarrieta (Department of Dermatology, Hospital Universitario de Móstoles, Madrid, Spain) provided the clinical annotation and validation.

Who funded the creation of the dataset?
This work was supported by the European Commission through the H2020 project WARIFA (Grant Agreement 101017385) and by the Spanish Government through the grants AAVis-BMR PID2019-107768RA and PID2023-149457OB (AEI/10.13039/501100011033).

1.2 Composition

What do the instances that comprise the dataset represent?
Each instance represents a single dermoscopic image, identified by its unique identifier in the source repository (e.g., ISIC identifier or filename), associated with a seven-dimensional binary vector indicating the presence or absence of each artifact category (hair, dark corners, gel border, gel bubbles, ruler markings, ink markings, and patches).

How many instances are there in total?
20,211 images in total: 19,000 from ISIC-2019, 1,011 from Derm7pt and 200 from PH2, corresponding to 141,477 image-level binary artifact labels (20,211 $\times$ 7).

Does the dataset contain all possible instances or is it a sample?
It provides annotations for the complete image sets of the three source repositories (not a sample).

What data does each instance consist of?
A source-image identifier plus seven binary labels (one per artifact category). The dermoscopic images themselves are not included, as the source repositories do not allow their redistribution; they must be obtained from the original datasets. Attention-based heatmaps produced by the C-Tran model are additionally provided for qualitative interpretability only and are not part of the labels.

Is there a label or target associated with each instance?
Yes: the seven per-image binary artifact presence/absence labels.

Is any information missing from individual instances?
The dermoscopic images are not redistributed due to the licensing terms of the source repositories; they must be obtained from the original sources and matched via the shared identifiers. No other information is intentionally missing.

Are relationships between individual instances made explicit?
Instances are linked to their source dataset and original image through unique identifiers. Dataset-level statistics (artifact prevalence, number of artifacts per image, and pairwise co-occurrence) are provided in the metadata.

Are there recommended data splits?
No artifact-specific splits are imposed. Users may adopt the original splits of each source repository as needed.

Are there any errors, sources of noise, or redundancies?
Some subtle artifacts (very small or partially visible structures) may be difficult to detect, and the annotations should be regarded as a high-quality reference rather than absolute ground truth. Labels were accepted automatically when the two independent detection strategies agreed. Disagreements were reviewed individually, together with a random subset of approximately 3% of the agreed labels in ISIC-2019

and Derm7pt. Agreement between the two strategies does not guarantee correctness, so the artifact-level disagreement rates (6.71% in ISIC-2019, 5.45% in Derm7pt, 4.07% in PH2) do not provide a direct estimate of the annotation error rate, as they cannot detect the cases in which both methods make the same error. On PH2, where every image was compared against an independently produced expert annotation, 5.51% of the automatically agreed decisions were inconsistent with that reference. Results of the manual validation are reported in Supplementary Table S3, and the per-artifact performance of the two automated components on PH2 in Supplementary Table S4.

Is the dataset self-contained, or does it link to/rely on external resources?
The annotations are self-contained, but they rely on the external source image repositories (ISIC-2019, PH2, Derm7pt), which remain publicly available.

Does the dataset contain data that is confidential or that might be considered sensitive?
No. It contains only artifact labels and image identifiers. The underlying dermoscopic images are anonymized in the source datasets, and no patient-identifiable metadata was processed.

1.3 Collection process

How was the data associated with each instance acquired?
Artifact labels were generated with a multi-phase, data-driven pipeline: *(i)* an ensemble of classical image-processing detectors (and deep learning segmentation models for hair), *(ii)* a transformer-based multi-label detector (C-Tran), and *(iii)* expert manual review of disagreements. Labels on which the automated approaches agreed were accepted directly; disagreements were reviewed by experts.

What mechanisms or procedures were used to collect/label the data?
Automated detection (described in the manuscript) followed by independent manual review by a computer-vision researcher and a board-certified dermatologist, who assigned the final binary labels.

Over what timeframe was the data collected/annotated?
The dataset was constructed between May and December 2025, a period covering both the automated (technical) artifact detection and the manual expert annotation and review. The resulting annotations were publicly released on Zenodo on 21 January 2026.

Were any ethical review processes conducted?
The dataset is built exclusively on publicly available, anonymized dermoscopic images released by the source repositories under their respective ethical approvals; no new human-subject data were collected.

Did you collect the data from individuals directly, or from third parties?
From third parties: the images originate from public dermoscopic datasets. Consent and acquisition were governed by the source repositories.

1.4 Preprocessing / cleaning / labeling

Was any preprocessing/cleaning/labeling of the data done?
Yes. Artifact labels were produced by the annotation pipeline described in the manuscript (classical detectors, segmentation models, C-Tran, and expert review). No modification was made to the source images.

Was the "raw" data saved in addition to the labeled data?
The released labels are the consolidated outputs. C-Tran attention heatmaps are additionally provided for qualitative inspection; intermediate detector masks are internal to the pipeline.

Is the software used to preprocess/label the instances available?
The full annotation pipeline (classical image-processing detectors, deep learning segmentation models, the C-Tran transformer, and the expert-review protocol) is described in detail in the Materials and Methods section of the accompanying manuscript, including all parameter settings, so that the procedure can be reproduced. The hair-segmentation code is publicly available[1], the C-Tran detector builds on the publicly available reference implementation of Lanchantin et al. (3), and the Double U-Net uses the implementation released by its original authors (4); and the classical artifact detectors are publicly available[2].

1.5 Uses

Has the dataset been used for any tasks already?
Yes. In the accompanying manuscript it is used to characterize artifact prevalence, artifact burden and pairwise co-occurrence across the three repositories.

What (other) tasks could the dataset be used for?
Artifact-aware CAD; development and benchmarking of artifact detection and removal (preprocessing) methods; study of dataset bias and spurious correlations; robustness and interpretability evaluation of skin-lesion classifiers.

Is there anything about the composition or collection that might impact future uses?
Artifact distributions differ markedly across the source datasets (e.g., dark corners dominate PH2, gel bubbles dominate Derm7pt), reflecting acquisition conditions; users should account for these differences. Labels for very small/subtle artifacts should be treated with caution.

Are there tasks for which the dataset should not be used?
It should not be used for clinical diagnosis, nor should the labels be interpreted as absolute ground truth for extremely small or partially visible artifacts.

1.6 Distribution

How will the dataset be distributed?
The artifact annotations are publicly available on Zenodo (`https://doi.org/10.5281/zenodo.18324962`). The dermoscopic images are not redistributed and must be obtained from the original repositories: ISIC-2019, PH2 and Derm7pt.

When will the dataset be distributed?
It is already publicly available.

Will the dataset be distributed under a copyright/license or terms of use?
The artifact annotations, metadata files and derived resources are released under the Creative Commons Attribution 4.0 International license (CC BY 4.0), copyright © The Authors. This license applies only to the

[1] `https://github.com/ai4healthurjc/hair-removal-aotgan-skin-lesions`

[2] `https://github.com/ai4healthurjc/dermartifactdb-classical-detectors`

annotations and derived resources; the original dermoscopic images are not covered by it and remain under the terms of their respective source repositories (ISIC-2019: CC BY-NC 4.0; PH2: research/educational only, no redistribution; Derm7pt: CC BY-NC-SA 4.0).

Do any IP-based or other restrictions apply?
Yes. Because the source repositories restrict redistribution and commercial use, only the artifact annotations (not the images) are redistributed here.

1.7 Maintenance

Who will be supporting/hosting/maintaining the dataset?
The authors, at Rey Juan Carlos University. The dataset is hosted on Zenodo.

How can the owner/curator be contacted?
vanesa.gomez@urjc.es; cristina.soguero@urjc.es

Will the dataset be updated? If so, how and by whom?
Corrections or extensions will be released as new versions on Zenodo, which preserves version history and DOIs. The DOI cited in this manuscript (`https://doi.org/10.5281/zenodo.18324962`) is the concept DOI, which always resolves to the latest version; readers requiring a fixed, citable version should use the version-specific DOI shown on the Zenodo record.

Is there a mechanism for others to extend/contribute to the dataset?
Yes: interested researchers may contact the authors, and contributions can be incorporated in future Zenodo versions.

2 COMPARISON WITH CNN BASELINES

To justify the choice of a transformer-based detector, C-Tran was compared against several CNN baselines for multi-label artifact detection on ISIC-2019, under the same five random train/test splits and evaluation protocol (see Table S1). C-Tran achieved the best performance across all aggregate metrics.

Table S1. Comparison of C-Tran against CNN baselines for multi-label artifact detection on ISIC-2019, under the same five random train/test splits and evaluation protocol. All CNN baselines use ImageNet-pretrained backbones with a multi-label classification head. Metrics: mean Average Precision (mAP), micro-averaged overall F1-score (OF1) and recall (OR), and macro-averaged class-wise F1-score (CF1). Values are mean ± std over the five test splits; best per metric in bold.

Model	mAP	OF1	OR	CF1
ResNet-101 (CNN)	0.9195 ± 0.0019	0.8504 ± 0.0037	0.8676 ± 0.0069	0.8570 ± 0.0038
DenseNet-121 (CNN)	0.9193 ± 0.0017	0.8530 ± 0.0031	0.8752 ± 0.0044	0.8586 ± 0.0043
EfficientNet-B0 (CNN)	0.9096 ± 0.0023	0.8427 ± 0.0031	0.8692 ± 0.0034	0.8468 ± 0.0033
**C-Tran (transformer)**	**0.9606 ± 0.0028**	**0.8719 ± 0.0025**	**0.9073 ± 0.0059**	**0.8900 ± 0.0016**

3 INTER-ANNOTATOR AGREEMENT

All artifact-level decisions submitted to expert review were labeled independently by the two annotators before any joint discussion. These comprise the decisions flagged by disagreement between the two automated approaches in ISIC-2019 and Derm7pt, together with the complete set of decisions of PH2, which was reviewed in full. Table S2 reports, for each artifact category, the percentage agreement and Cohen's kappa (5) between the two annotators prior to consensus, together with the number of decisions that required joint discussion. Consensus was reached in all cases.

Table S2. Inter-annotator agreement on the reviewed cases, before consensus discussion. The table reports, for each artifact category, the number of artifact-level decisions submitted to review, the percentage agreement and Cohen's kappa between the two annotators prior to any joint discussion, and the number of decisions that required joint discussion to reach consensus.

Artifact	Decisions reviewed	Percentage agreement	Cohen's κ	Cases requiring consensus
Hair	2,288	97.0%	0.94	69
Dark corners	2,650	98.0%	0.96	53
Gel border	634	85.0%	0.69	95
Gel bubbles	3,052	88.0%	0.76	366
Ruler markings	1,531	99.0%	0.98	15
Ink markings	351	96.9%	0.90	11
Patches	205	100.0%	1.00	0
Overall	**10,711**	**94.3%**	**0.88**	**609**

4 MANUAL VALIDATION OF THE AUTOMATICALLY ACCEPTED LABELS

Labels on which the classical ensemble and C-Tran agreed were accepted without individual review. To quantify their performance, a subset of these labels was inspected manually against the images. In ISIC-2019 and Derm7pt, a random sample of approximately 3% of the images (570 and 30 images, corresponding to 3,990 and 210 artifact-level decisions) was inspected. In PH2, the complete set of 1,343

agreement decisions was compared against an expert annotation produced independently of the annotation pipeline. Table S3 reports the number of corrected labels, their decomposition into false positives and false negatives, and their distribution by artifact category. All corrections were applied to the released annotations.

Table S3. Summary of the manual validation of automatically accepted labels across the ISIC-2019, Derm7pt, and PH2 datasets. The table reports the total number of decisions inspected, the total number of corrected labels and their decomposition into false positives (labels corrected from present to absent) and false negatives (labels corrected from absent to present), and the distribution of corrections by artifact type, expressed in both absolute counts (n) and percentages (%). All percentages are expressed over the number of decisions inspected in each dataset.

Artifact	ISIC-2019 (n)	ISIC-2019 (%)	Derm7pt (n)	Derm7pt (%)	PH2 (n)	PH2 (%)
Total decisions inspected	3,990	100%	210	100%	1,343	100%
Total corrected labels	110	2.76%	7	3.33%	74	5.51%
False positives	71	1.78%	5	2.38%	56	4.17%
False negatives	39	0.98%	2	0.95%	18	1.34%
Corrected labels by artifact type						
Hair	24	0.60%	3	1.43%	12	0.89%
Dark corners	30	0.75%	0	0.00%	7	0.52%
Gel border	5	0.13%	0	0.00%	17	1.27%
Gel bubbles	33	0.83%	3	1.43%	27	2.01%
Ruler markings	16	0.40%	1	0.48%	0	0.00%
Ink markings	2	0.05%	0	0.00%	11	0.82%
Patches	0	0.00%	0	0.00%	0	0.00%

5 EVALUATION OF THE AUTOMATED COMPONENTS ON PH2

The PH2 artifact labels were produced by manual expert annotation independently of the annotation pipeline, and therefore provide a reference against which the two automated components of Phase 2 and Phase 3 can be evaluated directly. Table S4 reports, for each artifact category, the sensitivity, specificity, precision, F1-score, and Cohen's kappa of the classical detector ensemble and of C-Tran with respect to that reference, computed over the 1,400 artifact-level decisions of the dataset. Sensitivity, precision, F1-score, and Cohen's κ are undefined for ruler markings and patches, which have no positive instances in PH2.

These results should be interpreted in the context of the class distribution in PH2, which is markedly imbalanced for several categories: dark corners are present in 184 of the 200 images, leaving only 16 negative instances, whereas ink markings occur in only 3. For such categories, a small number of individual errors can substantially affect the corresponding metrics, and specificity and precision should therefore be interpreted alongside the absolute instance counts rather than in isolation.

REFERENCES

[1]Gebru T, Morgenstern J, Vecchione B, Vaughan JW, Wallach H, Iii HD, et al. Datasheets for datasets. *Communications of the ACM* **64** (2021) 86–92.

[2]Wilkinson MD, Dumontier M, Aalbersberg IJ, Appleton G, Axton M, Baak A, et al. The fair guiding principles for scientific data management and stewardship. *Scientific data* **3** (2016) 1–9.

Table S4. Per-artifact performance of the classical detector ensemble and of C-Tran against the expert annotation of PH2 (200 images, 1,400 artifact-level decisions). Sensitivity, precision, F1-score, and Cohen's κ are undefined (–) for categories with no positive instances in the reference.

Artifact	Method	Sensitivity	Specificity	Precision	F1-score	Cohen's κ
Hair	Detector ensemble	0.920	0.840	0.775	0.841	0.733
	C-Tran	0.907	0.896	0.840	0.872	0.790
Dark corners	Detector ensemble	0.973	0.625	0.968	0.970	0.615
	C-Tran	0.951	0.875	0.989	0.970	0.689
Gel border	Detector ensemble	0.643	0.946	0.474	0.545	0.506
	C-Tran	0.571	0.903	0.308	0.400	0.340
Gel bubbles	Detector ensemble	0.897	0.765	0.663	0.762	0.610
	C-Tran	0.882	0.788	0.682	0.769	0.626
Ruler markings	Detector ensemble	–	1.000	–	–	–
	C-Tran	–	1.000	–	–	–
Ink markings	Detector ensemble	0.667	0.939	0.143	0.235	0.216
	C-Tran	0.667	0.949	0.167	0.267	0.249
Patches	Detector ensemble	–	1.000	–	–	–
	C-Tran	–	1.000	–	–	–

[3]Lanchantin J, et al. General multi-label image classification with transformers. *Proceedings of the IEEE/CVF Conference on Computer Vision and Pattern Recognition* (Nashville, TN, USA) (2021), 16478–16488.

[4]Jha D, et al. Doubleu-net: A deep convolutional neural network for medical image segmentation. *2020 IEEE 33rd International Symposium on Computer-Based Medical Systems (CBMS)* (Rochester, Minnesota, USA) (2020), 558–564.

[5]Cohen J. A coefficient of agreement for nominal scales. *Educational and psychological measurement* **20** (1960) 37–46.
